# Oxygen dynamics during *in vitro* seizures

**DOI:** 10.1186/1471-2202-13-S1-O20

**Published:** 2012-07-16

**Authors:** Yina Wei, Ghanim Ullah, Justin Ingram, Steven Schiff

**Affiliations:** 1 Center for Neural Engineering, Department of Engineering Science and Mechanics, Penn State University, PA 16802 USA; 2 Theoretical Biology and Biophysics, Los Alamos National Laboratory, NM 87545 USA; 3 Departments of Neurosurgery and Physics, Penn State University, PA 16802 USA

## 

Oxygen is an essential element for brain activity. The brain is a metabolic engine that requires 20% of the body’s metabolic energy, despite being only 2% of the human body mass [1]. Two thirds of brain’s metabolic energy is dedicated to supporting neural spiking activity. Much of the O_2_ dependent ATP metabolism in single neurons is used by energetic Na/K-ATPase pumps that transport 3Na^+^ outwards with 2K^+^ inward against their concentration gradients for each ATP hydrolyzed [2,3]. However, understanding the relationship between seizures and real-time oxygen dynamics has been restricted by current technical limitations. Computational models can offer insight to help understand the measurements from experiments.

We have performed experiments relating seizure activity at the cellular level with simultaneous real-time O_2_ microdomain measurements. In this paper, we build a mathematical neuron model that extends the Hodgkin-Huxley formalism containing leak currents for sodium, potassium and chloride ions, transient sodium currents, and delayed rectifier potassium currents. This neuron was embedded within an extracellular space and a simplified glia-endothelium system. The Na^+^ and K^+^ ion concentrations as well as extracellular oxygen density were continuously estimated. Hypoxia was modeled by reducing both neuron and glial Na/K-ATPase pump activities.

**Figure 1:**
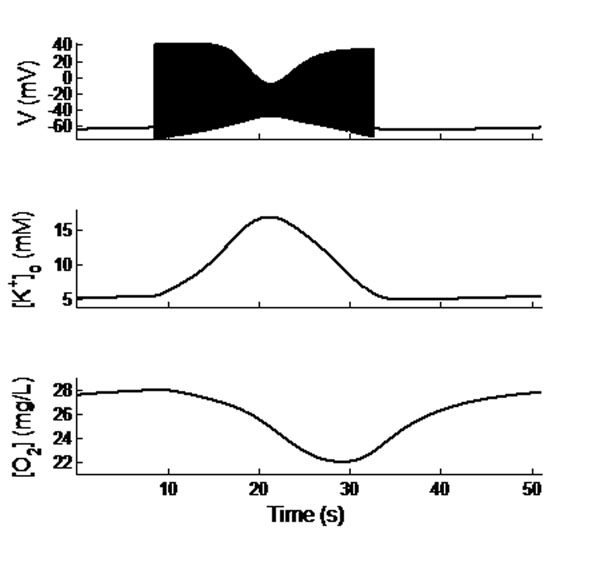
Membrane potentials (top trace), extracellular potassium concentration (middle trace), and oxygen density (bottom trace) from a single model neuron during a seizure event. The time course of [O_2_] debt is qualitatively similar to that observed experimentally.

During seizure events, the extracellular K^+^ and intracellular Na^+^ were increased, which further activated the Na/K-ATPase pump activity. Energy (ATP) and O_2_ demand were simultaneously increased. Therefore, local available [O_2_] decreased substantially during seizure events, and the apparent O_2_ debt substantially outlasts the intense electrical activity of a seizure, as shown in Figure 1. This result is consistent with experimental data [4]. We also observed that hypoxia alone can induce seizure like events, which occurs only in a narrow range of bath oxygen pressure, reflecting experimental observations. Lastly, we reproduced the interplay between excitatory and inhibitory neurons seen in experiments. Our model accounts for the different [O_2_] levels that we have observed during seizures in pyramidal cell layers vs. inhibitory (oriens lacunosum moleculare) cell layers. Our work suggests the critical importance of modeling extracellular ion concentration and oxygen dynamics to properly understand the underlying mechanisms behind seizure and related phenomena.
